# Liposomes and Their Therapeutic Applications in Enhancing Psoriasis and Breast Cancer Treatments

**DOI:** 10.3390/nano14211760

**Published:** 2024-11-01

**Authors:** Amal Ali Elkordy, David Hill, Mohamed Attia, Cheng Shu Chaw

**Affiliations:** 1 School of Pharmacy and Pharmaceutical Sciences, University of Sunderland, Sunderland SR1 3SD, UK; amal.elkordy@sunderland.ac.uk (A.A.E.); attia.attia@sunderland.ac.uk (M.A.); 2 School of Nursing and Health Sciences, Faculty of Health Sciences and Wellbeing, University of Sunderland, Sunderland SR1 3SD, UK; david.hill@sunderland.ac.uk

**Keywords:** breast cancer, psoriasis, combinational therapy, liposomes

## Abstract

Psoriasis and breast cancer are two examples of diseases where associated inflammatory pathways within the body’s immune system are implicated. Psoriasis is a complex, chronic and incurable inflammatory skin disorder that is primarily recognized by thick, scaly plaques on the skin. The most noticeable pathophysiological effect of psoriasis is the abnormal proliferation of keratinocytes. Breast cancer is currently the most diagnosed cancer and the leading cause of cancer-related death among women globally. While treatments targeting the primary tumor have significantly improved, preventing metastasis with systemic treatments is less effective. Nanocarriers such as liposomes and lipid nanoparticles have emerged as promising drug delivery systems for drug targeting and specificity. Advances in technologies and drug combinations have emerged to develop more efficient lipid nanocarriers to include more than one drug in combinational therapy to enhance treatment outcomes and/or relief symptoms for better patients’ quality of life. Although there are FDA-approved liposomes with anti-cancer drugs for breast cancer, there are still unmet clinical needs to reduce the side effects associated with those nanomedicines. Hence, combinational nano-therapy may eliminate some of the issues and challenges. Furthermore, there are no nanomedicines yet clinically available for psoriasis. Hence, this review will focus on liposomes encapsulated single and/or combinational therapy to augment treatment outcomes with an emphasis on the effectiveness of combinational therapy within liposomal-based nanoparticulate drug delivery systems to tackle psoriasis and breast cancer. This review will also include an overview of both diseases, challenges in delivering drug therapy and the roles of nanomedicines as well as psoriasis and breast cancer models used for testing therapeutic interventions to pave the way for effective in vivo testing prior to the clinical trials.

## 1. Introduction

Liposomes as nano-vesicular drug delivery systems have shown great success in their applications to target disease sites, and accordingly, there are literature review articles that focus, in general, on liposome applications. We aim to review liposomes encapsulated in single and/or combinational therapy to augment the treatment of breast cancer and psoriasis despite having different pathophysiological properties. Psoriasis, a representation of a chronic inflammatory skin condition currently without a cure, and breast cancer, representing a difficult-to-treat systemic disease where disease progression and responses to drug therapy, like many other cancers, are governed by the inflammatory processes in the body or the body’s immune system. Hence, for patients with either disease—psoriasis or breast cancer—and especially those with co-existing diseases, their symptoms and disease control, as well as side effect(s) of drug therapies, may increase or decrease as they may act on the same inflammatory pathways. This review is organized to include an overview of both diseases in terms of pathophysiology, current treatments and their challenges, relevant biological models for examining drug efficacy and safety, as well as the roles and benefits of nanomedicines. Then, a more focused discussion on the application of nano-particular systems based on biological evidence of liposomes entrapped with either single or combinational therapy to demonstrate drug efficacy and safety, especially where combination therapies were applied topically and parenterally in psoriasis and breast cancer, respectively, will be presented.

## 2. Psoriasis

Psoriasis is a chronic autoimmune skin disease. The pathogenesis of psoriasis involves the interactions among keratinocytes, immune cells and vascular endothelial cells that are triggered by genetic or/and external factors. Epidermal hyperplasia resulting from the proliferation and aberrant differentiation of keratinocytes, vascular alteration with dilated blood vessels that leads to the infiltration by inflammatory cells, as well as cytokines production in the epidermis and dermis, are featured in psoriatic skin [1]. There is also an increase in the number of dividing keratinocytes in the basal layer of the epidermis, hyperkeratosis and parakeratosis, as well as the loss of cornified layer that is featured by acanthosis [2] and accelerated skin desquamation in stratum corneum [3]. These structural defects compromise the epidermal barrier function of psoriatic skin [4]. In the psoriatic lesions, immune cells secrete proinflammatory cytokines to initiate, maintain and augment the inflammatory responses after infiltrating the dermis and epidermis [5]. Apart from dendritic cells like CD11c+ myeloid dendritic cells (MDC) and Langerhans cells, CD4+ T and CD8+ T cells infiltrate the upper dermis and epidermis, respectively. MDCs release several mediators that participate in activation, polarization, expansion of T cells and keratinocyte abnormalities [5]. Interaction between MDC and T cells to co-stimulate the excretion of numerous inflammatory cytokines in a sustained manner is also associated with the effect of antimicrobial peptides from keratinocytes in psoriatic lesions [1]. Keratinocytes that are activated also release chemokines, promoting leukocyte infiltration to the skin area to exacerbate inflammatory responses in psoriatic lesions. This crosstalk between keratinocytes and immune cells also creates an inflammatory loop, leading to the persistence or exacerbation of psoriasis plaques [6].

**Figure 1:**
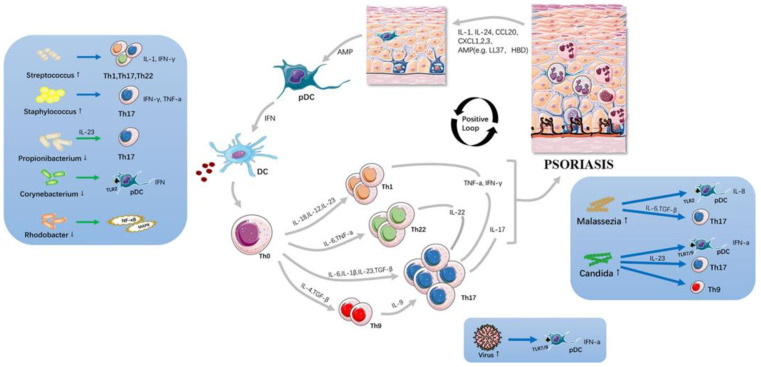
Pathogenesis of psoriasis showing cross talk and interactions among cells of the immune system, keratinocytes and skin microbiome at the lesional site with the release of different cytokines or chemokines. The altered skin microbiota disrupts the skin barrier and acts on the innate immune system that activates the inflammatory cascades. Skin microbiota is considered a key trigger in the psoriatic inflammation loop. Figure adapted from [8].

Microorganisms can reside in different parts of the body’s skin as they adopt special mechanisms to survive against antimicrobial peptides and the constructed cornified barrier of keratinocytes [7,8]. The normal skin microbiome is made of both beneficial and potentially harmful microbes, of which *Firmicutes*, *Actinobacteria*, *Bacteroidetes* and *Proteobacteria phyla* are the common species. They regulate resident immune cells and keratinocytes that participate in inflammation responses. In contrast to normal skin, microbial colonization of psoriasis lesions is mainly confined to the epidermis. Pruritus can induce scratching that breaks the skin to form a wound, which allows epidermal colonizers and other microbes to reach the deep dermis or even peripheral blood where these microbes, when in contact with immune cells, stimulate innate and adaptive responses [9,10,11]. This can trigger dysbiosis of skin microbiota to worsen psoriatic skin conditions. Figure 1 depicts the pathogenesis of psoriasis and the involvement of skin microbiota. Hence, the classical characteristics of psoriatic skin lesions, which hinder penetration and percutaneous absorption of many effective agents, along with changes in skin microbiota, the crosstalk between immune cells and skin cells that changes the immune responses in the dermis and epidermis, necessitate the development of novel drug delivery systems.

### 2.1. Mechanisms Underlying Psoriasis and Cell Biology Models for Psoriasis

**Figure 2:**
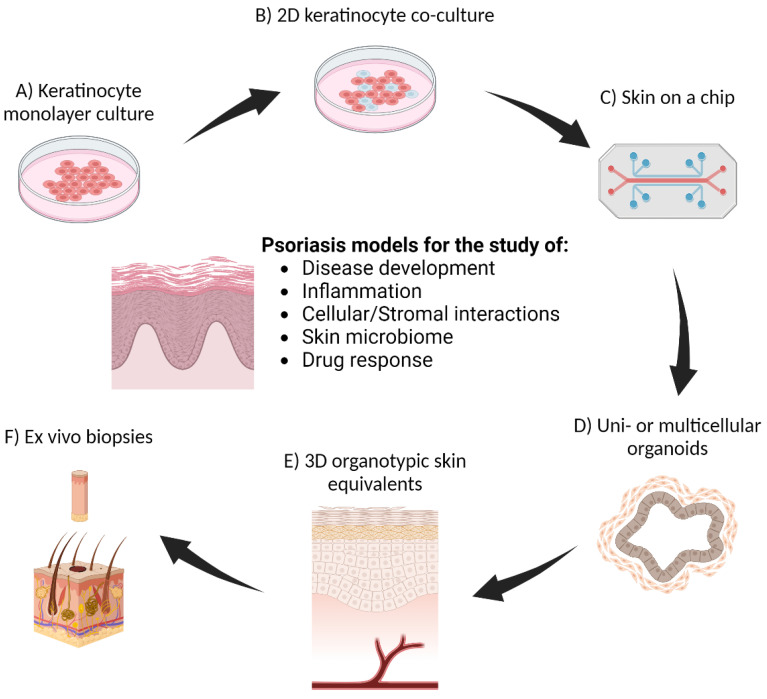
Cell biology models for the study of psoriasis. (**A**) Keratinocyte monolayer culture is a technically simple and versatile model for most applications. (**B**) Keratinocyte co-culture models with other cell types, including fibroblasts, endothelial cells and immune cells, are also performed in 2D monolayers but are more complex, requiring media optimization for multicellular culture. (**C**) Organ-on-a-chip models use microfluidics to maintain a consistent homeostatic microenvironment for continuous skin culture. (**D**) Organoids are another versatile model that can self-assemble or be bio-printed with keratinocytes only or multiple cell types, with or without stromal matrix added. (**E**) Full-thickness organotypic skin models are the most physiologically relevant models but require the most time and expertise to perform. (**F**) Biopsy skin explants can provide patient-specific drug response data, but sample size is a limiting factor in their use.

It is crucial to investigate the mechanism of psoriasis and to study the cellular and molecular mechanisms underlying psoriasis. Monolayer tissue culture models are widely used as they offer a controlled environment to investigate various aspects of psoriasis pathophysiology, including keratinocyte proliferation, differentiation, and the inflammatory response (Figure 2). Keratinocyte monolayer cultures created from isolated human skin or immortalized keratinocyte cell lines (such as HaCat or CCD1106 cells) are used to study the abnormal proliferation and differentiation of keratinocytes. Such models have been valuable for assessing the effects of various cytokines, such as IL-17, IL-22, and TNF-α, which are known to be involved in the inflammatory response in psoriasis [12]. The addition of these pro-inflammatory cytokines to keratinocytes has been shown to induce a psoriatic-like phenotype [13], which can subsequently be used to screen potential anti-inflammatory drugs and to understand the molecular mechanisms driving psoriasis. To better mimic the complex interactions between different cell types in the skin, co-culture models have been developed, which involve growing keratinocytes with other cell types, such as fibroblasts, immune cells or endothelial cells [14]. Keratinocyte and T-cell co-cultures have been used to investigate the role of T-cell-derived cytokines in psoriasis, which has improved our understanding of the crosstalk between keratinocytes and immune cells that are responsible for the development of psoriatic lesions [15]. Human skin cells have even been incorporated into innovative ‘skin-on-a-chip’ approaches, which involve the use of microfluidic systems that maintain these multicellular models for extended periods, allowing for detailed studies of skin physiology and pathology under dynamic conditions [16].

Scaffold-based 3D models, using either natural or synthetic scaffolds to support the growth and interaction of skin cells, have been used to create structures that closely resemble the mechanical, structural, and biochemical properties of native skin [17]. Scaffold-free models, often based on self-assembling or bio-printed spheroids, offer a high-throughput option for studying skin tissue dynamics and drug testing, with some advanced versions even including smooth muscle and endothelial cells to mimic vasculature, enhancing the physiological relevance of the models [18]. These models are useful for understanding the mechanisms of skin–cell interaction and the effects of treatments on tissue microenvironments.

Advances in 3D printing have facilitated the development of bio-printed skin models that closely mimic natural skin structures [19,20]. Such technology builds upon previous skin models that incorporate various cell types and bioactive molecules to replicate the physiological and biochemical properties of human skin [21,22]. These models are particularly beneficial for studying drug interactions and disease mechanisms in a physiologically relevant tissue-like environment. Immune activation within skin equivalents has been observed via cytokine production in response to immune-sensitizing compounds [23], while the integration of immune cells or inflammatory cytokines enables these models to mimic complex biological interactions and inflammatory processes observed in psoriasis [24].

### 2.2. Current Treatment for Psoriasis

**Figure 3:**
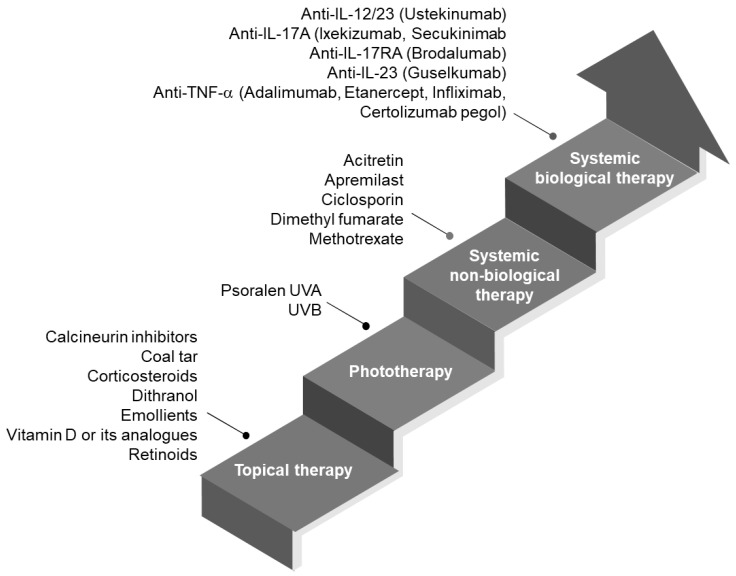
Psoriasis treatment ladder with representative drug classes updated from [25].

Psoriasis is a chronic skin disorder that is currently without cure. The main therapies aim at relieving symptoms to control the disease state. Depending on the degree of disease severity, several therapies have been prescribed. Figure 3 shows the psoriasis treatment options where a combination of treatments may use multiple “steps” of the ladder.

For mild to moderate psoriasis, where small areas of skin surfaces are affected, this can be managed with standard topical therapy. In moderate to severe psoriasis, where large areas of skin surfaces are affected and where severe disease subtypes exist or with exhibition of disease skin in the scalp or sensitive body sites, topical therapy in combination with biological agents, systemic therapy or phototherapy is recommended [26]. In addition, emollients may be necessary to alleviate dry and scaly skin, while antimicrobial agents are prescribed to control infected skin. Common agents for topical applications comprise small molecules that modulate the immune system of the skin. Tacrolimus and pimecrolimus bind immunophilins and cyclophilins to inhibit calcineurin activation [27]. Corticosteroids with different degrees of potency prevent phospholipase A2 relapse and amplify anti-inflammatory cytokine gene expression and are most commonly prescribed [28]. Vitamin D analogs bind to vitamin D receptors to interfere with dendritic cell maturation and T-cell activation. Dithranol, an anthralin that blocks keratinocyte proliferation and T-cell activation, is recommended for scalp and plaque psoriasis. Tazarotene binds retinoic acid and retinoid X receptors on keratinocyte cells and can modify inflammatory genes. Coal tar, an old remedy, is postulated to suppress gene synthesis to inhibit keratinocyte proliferation. Salicylic acid is a keratolytic agent that is effective in softening and removing psoriatic scales. Combination therapy of corticosteroids and other topical agents mentioned has been prescribed to patients to better control the disease state, alleviate symptoms and reduce side effects. Topical therapies are presented in several forms that either incorporate single or multiple anti-psoriatic agents to improve efficacy and compliance. Cream and ointment are the most widely manufactured, while other dosage forms such as gels, medicated liquids, foams, paste, medicated plaster and impregnated dressing are also available [29]. Systemic therapies for psoriasis treatment are administered either via oral or parenteral route [30,31]. Though they are effective, as drugs reach the systemic circulation, patients are put at a greater risk of experiencing unwanted side effects. Oral therapies include retinoid analogs, methotrexate, ciclosporin A, dimethyl fumarate and tyrosine kinase inhibitors. They are presented as tablets or capsules with known effects on the immune system; for example, methotrexate inhibits dihydrofolate reductase to halt the proliferation of keratinocytes and immune cells, e.g., ciclosporin inhibits calcineurin phosphatase activity, T-cell activation and keratinocyte proliferation. Parenteral therapies are based on several biologics that are either recombinant monoclonal antibodies or fusion proteins that exert their effects by blocking specific cytokines or cytokine receptors that trigger an inflammatory response in psoriatic skin. They are highly effective treatments, especially for severe disease states. Most of the preparations are manufactured as sterile liquid concentrates that can be self-injected via the subcutaneous route.

### 2.3. Issues with Current Psoriasis Treatments

Psoriasis is a chronic disease where the management remains challenging as there is no curative treatment. Not only is there an involvement of the immune system, but the complex etiologies of psoriasis can be difficult to handle, especially with the presence of serious wounds. The safety profiles of many promising agents are related to immune system suppression and damage of key organs when these agents are given at high doses as well as on repeat usage. For example, patients are at a greater risk of hepatotoxicity and more cardiovascular and gastrointestinal tract issues that require regular monitoring and dose normalization when ciclosporin is prescribed [32]. For topical therapy, variable skin absorption due to limited permeation from conventional dosage forms has limited drug availability at psoriatic lesions. Furthermore, poor retention and/or lack of sustained release of the drug to maintain its effect at psoriatic lesions reduces overall efficacy. Poor patient adherences are also evidenced for certain agents, for example, coal tar or dithranol, which cause skin staining and discoloration [33]. There are also issues with the physicochemical properties of some drugs, for example, dithranol has low solubility, limited permeability and stability, while biologic is too large to permeate the skin and must be delivered via the parenteral route, which is invasive in nature [34,35,36].

## 3. Breast Cancer

Breast cancer is the second cause of mortality in females and one of the most common types of cancer among other types [37]. Globally, breast cancer accounts for one in eight cancer diagnoses [38]. Breast cancer is known to be a collection of diseases characterized by different biological subtypes that exhibit distinct molecular profiles and clinicopathological properties. The histopathological classification of breast cancer can be broadly divided into two groups: in situ carcinoma and invasive (infiltrating) carcinoma. Breast carcinoma in situ can be further grouped into ductal carcinoma in situ (DCIS) and lobular carcinoma in situ (LCIS). LCIS is believed to stem from the presence of atypical lobular hyperplasia, while DCIS lesions are most commonly observed in the mammary ducts [39]. In contrast, molecular categorization involves grouping tumors based on their reaction to growth hormones and hormones. The estrogen receptor (ER), the progesterone receptor (PR) and the Human Epidermal Growth Factor Receptor 2 (HER2) are the most important clinical receptors in this area. Based on immunohistochemical characteristics and the hormone receptor (HR) status of the disease physiology, breast cancer tumors are, hence, categorized into four primary subtypes, including HR+/HER2+ tumors that react to estrogen or progesterone and possess HER2 receptors, HR+/HER2- tumors that react to estrogen or progesterone but lack HER2 receptors, HR-/HER2+ tumors that express HER2 receptors but do not possess hormone receptors, and HR-/HER2- tumors or triple-negative breast cancer (TNBC) [37,39].

### 3.1. Hallmarks of Breast Cancer

Cancer cells, in contrast to normal cells, engage various mechanisms to evade apoptosis. The intrinsic pathway of apoptosis has been shown to be frequently disrupted in cancer cells and is closely regulated by cellular metabolism. While oncogenes and tumor suppressors reprogram tumor metabolism, microenvironment or therapy-imposed stresses can additionally rewire metabolism, creating new metabolic dependencies that can play a crucial role in tumor sustainability and proliferation.

Apoptosis is a mode of programmed cell death essential for maintaining tissue homeostasis by elimination of unwanted, superfluous, and damaged cells. Apoptosis occurs discretely in individual cells of our body and is a highly regulated energy-dependent process. Deregulation of apoptosis is involved in the pathogenesis of several diseases like neurodegenerative conditions, which involve excessive apoptosis, as well as cancer, which, in contrast, is characterized by accumulation of cells exhibiting insufficient engagement of the apoptotic machinery and evasion of apoptosis [40]. There are two primary pathways that lead to apoptosis: the intrinsic pathway of apoptosis and the extrinsic pathway of apoptosis. Both pathways result in the activation of cysteine aspartyl-specific proteases or ‘caspases’, which are the final effectors of apoptosis and cleave several proteins leading to cell death [40]. The extrinsic pathway can be engaged by activation of death receptors of the tumor necrosis factor (TNF) superfamily, such as Fas (Apo/CD95), TNF Receptor 1 (TNFR1) and TNF-related apoptosis-inducing ligand (TRAIL) receptors, etc., located on the cell surface, by binding with their specific ligands [41,42]. On the other hand, induction of the intrinsic (mitochondrial) pathway is primarily regulated by the B cell lymphoma (BCL-2) family of proteins and is activated by internal stress sensors in response to cellular stresses like nutrient deprivation, DNA damage, hypoxia, etc. Detachment of cells from the extracellular matrix can also induce a form of apoptotic cell death called ‘anoikis’, which acts to control the growth and re-attachment of detached cells to a different matrix [40]. Since tumor formation is a multistep process, normal cells evolve progressively to the neoplastic stage, and along the way, they acquire particular capacities and microenvironments that enable them to become tumorigenic. These microenvironment plays a catalytic role in breast cancer progression and survival [43].

### 3.2. Cell Biology Models in Breast Cancer

Several cell biology models are commonly used to investigate breast cancer, providing crucial insights into the disease’s mechanisms, progression, and potential treatments. Breast cancer research heavily relies on cultured cell lines derived from human tumors. These cell lines are used to study cancer cell biology, genetics, and response to drugs. In addition, these models can be used to examine the effects of inflammatory cytokines within the breast tumor microenvironment [44]. Inflammatory cytokines such as TNF-α promote inflammation that leads to excessive skin cell proliferation in psoriasis; it also supports tumor growth and the inflammatory environment necessary for cancer progression. While IL-17/IL-23 in psoriasis drives immune cell activation, leading to chronic skin inflammation, this same pathway has been implicated in breast cancer, as IL-17 contributes to tumor growth and metastasis by attracting immune cells that support tumor expansion. Some well-known breast cancer cell lines include MCF-7 (estrogen receptor-positive), MDA-MB-231 (triple-negative), and SK-BR-3 (HER2-positive). To better mimic the tumor environment, as with studies that model psoriasis, researchers use 3D culture systems, such as spheroids and organoids [45]. These models provide a more physiologically relevant context, allowing the study of cellular interactions, tumor morphology, and drug penetration in a controlled environment. Novel technology has made use of microfluidic devices to create an ‘organ-on-a-chip’ model of breast cancer, which allows for higher throughput study of tumor–stroma interactions, metabolic pathways and drug response [46].

Human breast cancer cells can also be implanted into immunocompromised mice to form xenograft tumors, which allows for the study of tumor growth, metastasis and response of human cancer cells to therapy in an in vivo environment. A further development of this model involves the use of cancer cells directly from a patient to create patient-derived xenografts (PDXs) that are implanted into immunocompromised mice [47]. PDX models maintain the histological and genetic characteristics of the original tumor, making them valuable for personalized medicine research. Alternatively, genetically engineered mouse models (GEMMs) have specific genes knocked out or overexpressed to develop murine breast cancer, which helps in understanding the role of specific genes in cancer development and progression [48].

### 3.3. Current Treatment for Breast Cancer

The main types of treatment for breast cancer are surgery (mastectomy), radiation therapy (RT), chemotherapy (CT), endocrine (hormone) therapy (ET), and targeted therapy [49,50]. The focus of this review will be on CT; information on other therapies can be found elsewhere, for example [51,52]. The chemotherapeutic agents used for breast cancer treatment cause adverse drug reactions in addition to their therapeutic outcomes, and such adverse reactions discourage patient adherence to the therapy. Hence, the pre- and post-treatments to cope with such indications must be in line with the standard treatment recommendations [53]. The benefit from CT is more pronounced in ER-negative tumors. CT is recommended in the vast majority of TNBC, HER2-positive breast cancers and in high-risk luminal tumors [39]. The therapeutic options and the cocktails of drugs are often applied to each patient in a personalized manner since every tumor phenotype is different according to the individual’s pathology. Among the numerous chemotherapeutic cocktails implemented in a combinational way for personalized treatment by targeting different cellular pathways, the family of anthracycline antibiotics (i.e., doxorubicin, epirubicin and daunorubicin) and taxanes (i.e., paclitaxel, docetaxel) are the most common chemotherapeutic standards for breast cancer treatment, and they are given in combination or in sequence for a period of 18–24 weeks, prior to or post-surgery [54].

### 3.4. Issues with Current Breast Cancer Treatment

With metastatic disease being a major cause of death among those who succumb to breast cancer, the long latency period between initial treatment and potential recurrence suggests the tumor interacts with and alters the host’s systemic environment, aiding disease progression. Significant advances in recent years have revealed important insights into the genetic basis and risk factors for breast cancer, which are aiding early detection and development of more effective treatment options. A comprehensive analysis focused on identifying target genes within breast cancer susceptibility loci, utilizing data from genome-wide association studies (GWAS), which highlighted the role of functional genomics in understanding cancer risk variants [55]. However, the utility of gene-expression profiling in early breast cancer is still unclear as there is little evidence that these tests can be used to direct treatment decisions to improve patient outcomes [56]. Studies have explored the evolutionary history of metastatic breast cancer, providing evidence supporting both linear and parallel progression models [57]. Population-based studies have reaffirmed the significantly increased risks of breast and ovarian cancer for carriers of BRCA1 and BRCA2 mutations, emphasizing the importance of genetic screening for at-risk individuals [58]. Interestingly, disruption of the circadian rhythm, for example, by night-shift work, is an often-underappreciated potential risk factor for breast cancer, suggesting the importance of maintaining regular sleep patterns as part of cancer prevention strategies [59]. Innovations in screening techniques have been a major focus of recent research. Notably, the United States Preventive Services Task Force recommended lowering the age for breast cancer screening from 50 to 40, which aims to increase early detection rates, potentially saving more lives [60]. Furthermore, technologies like artificial intelligence [61] and liquid biopsies [62] are becoming integral in detecting early-stage cancers more effectively and safely.

The FDA has approved several new anticancer therapeutics, such as the selective estrogen receptor inhibitor Elacestrant, for certain types of breast cancer. This drug, among others, reflects a trend towards more personalized medicine approaches [60]. Additionally, the development of treatments such as T-cell-engaging bispecific antibodies and antibody-drug conjugates marks a significant step forward in immunotherapy and targeted therapy [63].

## 4. Roles of Nanomedicines with Inclusion of Combinational Therapy for Treating Psoriasis and Breast Cancer

### 4.1. Nanomedicines in Psoriasis

Compared to the stratum corneum, the viable layers beneath it, the epidermis and dermis, are more hydrophilic. This is why it is tricky to construct a formulation that will be able to penetrate across the different skin layers. Drug delivery systems that can be used to achieve this are nano-carriers such as liposomes, micelles, nanoparticles and nano-colloidal silica with the addition of gelling agents to achieve greater skin retention. They enhance drug availability when applied topically to provide a better therapeutic effect, which is partly due to their small sizes that increase the surface area of exposure. Entrapping drugs in nanocarriers can modulate the hydrophilic and lipophilic balance of drugs with either relatively high or small partition coefficients to increase their permeability across the stratum corneum and to allow drug penetration through the thick outer barrier in psoriatic skin to reach the viable parts where immune responses should be controlled. Drugs with relatively poor stability or large in molecular weight or macromolecules such as immunosuppressants, biologics or gene therapy as well as those exhibiting low solubility or that are very hydrophilic, can be entrapped in nanocarriers to improve their overall percutaneous absorption. The bulk and surface properties of nanocarriers can be modified to achieve better drug targeting for greater accumulation of drug molecules at lesion sites. All these improved features that can be brought by the application of nanocarriers can lead to a further reduction in dose, dosing frequency, dose-dependency and controlled drug release from such systems to exert better therapeutic outcomes while lessening unwanted side effects.

There have been several reviews that discuss the application of drug delivery and nano-based systems to deliver drugs for psoriasis in the past—refer, for example, to [36]—however, with no focus on the recent combinational therapy within nano-based delivery systems. The type of vehicles used to deliver a given drug can affect the permeation of the drug on the psoriatic skin to increase or decrease its therapeutic effect. Within the field of nanomedicine, the nanocarriers can be classified according to the matrix components used to fabricate these carriers, which are either organic materials such as lipids or polymers or inorganic materials that can be sourced naturally or chemically synthesized. For lipid-based carriers, they are divided into vesicular or particulate systems such as liposomes, solid lipid nanoparticles, nanostructured lipid carriers or lipospheres and emulsion-based systems. For polymeric nanocarriers, they may include self-assembled, particulate and capsular structures, e.g., micelles, nanoparticles, dendrimers, nanospheres and nano-capsules that were produced from synthetic or biopolymers.

### 4.2. Nanomedicines in Breast Cancer

**Figure 4:**
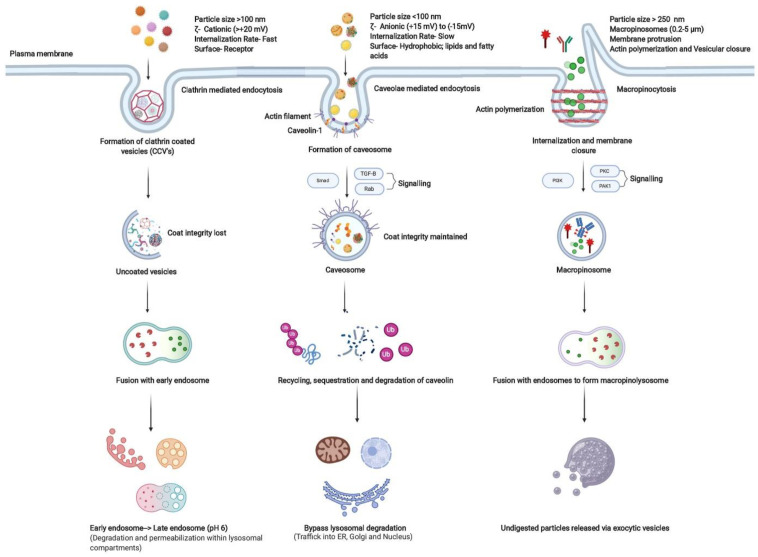
Schematic representation of endocytic pathways involved in the internalization of amphiphilic copolymers and the associated factors modulating their uptake pathways and intracellular fate. Figure adapted from [64].

The ability to deliver drug molecules specifically and safely to the selected cell types at therapeutically effective concentration invokes a major challenge in drug delivery. One of the possible approaches to ensure the safety, specificity and efficacy of drug molecules is the use of nanomedicines or nanoparticulate agents in breast cancer as well. The major goals of nanomedicine development include the creation of improved formulations with targeting ability and controlled drug release along with reduced toxicity and the ability to bypass biological barriers and reach the target site [64]. The interactions of nanovesicles such as liposomes with cells are dependent on various factors, including liposome size, morphology, charge, lipid composition, bilayer packaging, surface characteristics and the presence of surface ligands, among others [65]. Based on these features and the tumor physiology, scientists fabricated liposomal carriers with adaptable characteristics for efficient drug delivery. The amphiphilic nature of the phospholipids enables liposome engulfment by cells via phagocytosis and/or endocytosis mechanisms or receptor-mediated endocytosis (Figure 4) [66].

### 4.3. Combinational Therapy

Both psoriasis and breast cancer are complex diseases; hence, the combination of two or more drugs will be beneficial compared to monotherapy because this will help reduce the side effects of drugs and drug resistance and fulfill some of the unmet clinical needs. This method proved successful in tackling challenges with complex diseases. Accordingly, in the following parts, we will review the application of vesicular systems with a focus on the recent advances in liposomes loaded with combinational therapy to tackle two diseases: psoriasis and breast cancer.

## 5. Liposomes Formulations for Drug Delivery

**Figure 5:**
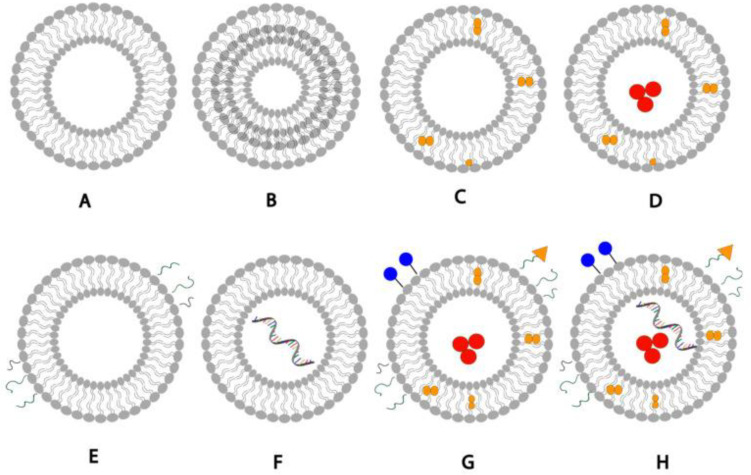
Schematic representation of structure of liposomal drug delivery systems: (**A**) unilamellar liposome, (**B**) multilamellar liposome, (**C**) liposome loaded with a hydrophobic drug, (**D**) liposome loaded with a hydrophobic drug in the bilayer membrane and a hydrophilic drug in the aqueous core, (**E**) pegylated liposome with surface PEG polymer chains, (**F**) liposome loaded with mRNA, (**G**) liposome with a surface-conjugated drug, targeting ligands and PEG, hydrophilic and hydrophobic drugs, (**H**) liposome with a surface-conjugated drug, targeting ligands, PEG polymer chains, hydrophilic drugs, hydrophobic drugs, mRNA-loaded. Figure adapted from [67].

Conventional liposomes comprise lipid bilayers made of phospholipids, cholesterol and stabilizer, which enclose an aqueous core. Both the lipid bilayer and the aqueous space can entrap compounds of different physicochemical properties. Liposomes are sub-classified according to their surface charges and the number of lipid bilayers found within the vesicles. They are produced by well-established methods that have been previously reviewed (refer, for example, to [67,68,69]). There are several commercial products based on liposomes, for example, Myocet^®^ (liposomal doxorubicin used in treating metastatic breast cancer). Figure 5 shows a schematic representation of the structure of liposomal drug delivery systems.

### 5.1. Liposomes and Psoriasis

Over the years, there has been an increasing number of papers investigating the application of conventional liposomes for psoriasis; this has led to the filing of several patents [70]. Loading of anti-psoriatic agents into liposomes resolves several issues that are related to the unfavorable intrinsic properties of drugs, such as poor solubility, chemical instability, unfavorable partition coefficient and high molecular weight. The size of the vesicle and its distribution are identified as essential properties to ensure successful drug delivery for nanocarriers, such as via the topical route, especially where transcutaneous absorption is necessary.

Most of the liposomes containing anti-psoriatic agents are produced by the conventional thin film hydration (TFH) method that undergoes further size reduction by sonication, then membrane extruded to achieve a nano-size range with a narrow size distribution and an acceptable entrapment efficiency. In addition, applying liposomal formulations has overcome the skin barrier and has led to enhanced skin retention, drug penetration and local availability, which have been confirmed by using either relevant cell culture and/or ex vivo skin to show drug permeation to the dermis. In addition, a reduced level of cytokine storm and symptom alleviations for psoriatic-like lesions based on biochemical, histological and visual examinations were proven by imiquimod-induced psoriatic animal models in many cases, confirming drug molecules that were released from liposomes had transported into viable portion of the skin.

To date, the majority of these liposomes have successfully loaded with a single anti-psoriatic agent regardless of their physicochemical properties, and the recent development of liposomal formulations entrapped with a single anti-psoriatic agent is discussed below.

In Lu et al., 2024, the therapeutic effect and mechanism of action of glabridin-loaded liposomes were studied in an imiquimod-induced mice model where drug-containing liposomes improved symptoms of psoriasis by downregulating mast cell infiltration, reducing Psoriasis Area and Severity Index (PASI) score and decreasing IL-23 and STAT3 mRNA expression. Furthermore, expression of TNF-α, IL-17 and IL-22 were reduced based on immunohistochemistry and enzyme-linked immunosorbent assay [71]. To improve the permeation of curcumin, an ionic liquid was first produced from curcumin succinic anhydride and betaine before entrapment into liposomes using an ethanol injection method [72]. In the excised mouse skin model, liposomes penetrated the stratum corneum, delivering curcumin to the epidermis and dermis. Uptake of drug molecules by human immortalized keratinocytes reduced TNF-α, IL-1β, IL-17A, IL-17F and IL-22 expression with an increased expression of collagen-I also observed that warrants further tests with either an in vivo or an equivalent 3D psoriatic cell culture model to confirm its anti-psoriatic effect [72]. Liposomal spherical nucleic acids to target IL-17A receptors were tested in imiquimod-induced mouse and 3D draft models [73]. These liposomes loaded with antisense oligonucleotides increased expression of IL-17 and other psoriatic biomarkers and were trialed for the treatment of mild-to-moderate psoriasis.

Transcutaneous drug transportation can be further aided by dispersing liposomes in a suitable gelling agent that creates an occlusive film to hydrate the stratum corneum. In Javia et al., 2022, omiganan, an antimicrobial peptide, was loaded onto liposomes by reverse-phase evaporation. After subjecting liposomes to probe sonication, they were incorporated into carbopol 943P gel. Liposomal gel reduced IL-6 and TNF-α levels in the imiquimod skin model, which was confirmed by an improved drug permeation in ex vivo skin assay when compared to standard gel and lotion loaded with the peptide drug [74]. Bexarotene, a retinoid X receptor agonist with high log P and poor solubility that limits its transcutaneous permeation, was entrapped in TFH-based liposomes that were probe sonicated before loading onto methylcellulose gel. Liposomes showed slow drug dissolution profiles and an enhancement in drug permeation and transdermal flux that was demonstrated by the presence of loaded fluorescence dye in a deeper layer of the dermis in the ex vivo skin test. In the imiquimod-treated mice model, liposome gel reduced levels of IL-17, 23, 22 and TNF-α as well as scaling and skin inflammation [75]. In Walunj et al., 2020, cationic liposomes loaded with ciclosporin were prepared using the ethanol injection technique before converting them into a liposomal gel that exhibited a shear-thinning property. In the imiquimod-induced skin model, levels of IL-17, IL-22 and TNF-α, as well as PASI score, were reduced when compared to the untreated group [76]. Liu et al. (2024) produced a sustained-release arsenic trioxide-loaded liposomes gel using a zinc acetate gradient method. Liposomal gel reduced PASI score and levels of IL-6 and TNF-α in the imiquimod-induced mice model, and this gel was superior to tacrolimus therapy [77]. Brilaroxazine containing TFH-based liposomes was produced by hydrating the lipid film in maltodextrin solution before converting it into a lipogel to overcome limited solubility and high log P of the drug. Using the imiquimod-induced psoriatic model, the drug-containing lipogel had lower Baker and PASI scores than the imiquimod-induced control group. Lower levels of Ki-67, a proliferative marker, and TGF-β, a pro-inflammatory, were also reported [78]. Topical quercetin therapy has limited efficacy as it tends to recrystallize when prepared in conventional semisolid dosage forms. Carbomer gel loaded with quercetin containing TFH-based liposomes that have been sonicated, membrane extruded and modified with hydroxypropyl-β-cyclodextrin were prepared [79]. Enhancement in drug permeation and penetration into the excised rat skin were observed as the presence of cyclodextrin has increased the molecular interaction between liposomes and skin matrix lipids. In the imiquimod-induced model, the cyclodextrin-modified liposomal gel has lowered PASI score, suppressed skin thickening and downregulated TNF-α, IL-17A and IL-1β levels when compared to unmodified liposomal gels [79]. In Xi et al., 2022, after probe sonication and extrusion to obtain narrow size distribution, TFH-based mannosylated liposomes loaded with celastrol were used to target dendritic cells found beneath the stratum corneum after intradermal injection [80]. The modified liposomes exhibited a gradual drug release profile with a greater uptake into the dendritic cell line that prevented the surface expression of costimulatory factors, confirming the anti-maturation effect of released celastrol. There were also decreased levels of IL-17 and IL-23 in the imiquimod-induced mouse model when compared to celastrol liposomes and free drug preparation. Curcumin liposomes and peptide-modified curcumin liposomes were prepared by Yu and coworkers and showed high cellular uptake that inhibited cell proliferation [81]. In the imiquimod-induced mouse model, peptide-modified curcumin liposomes reduced skin inflammation and epidermal thickness more than curcumin liposomes with a reduction in levels of IL-17A, IL-17F and TNF-α due to interaction between peptide and Na+/K+-ATPase beta subunit. Hence, functionalizing liposome surface has improved skin penetration and transcutaneous absorption of anti-psoriatic agents. Also, incorporating natural permeation enhancers to liposomes that modulate the hydration state of either drug or/and skin has further helped to deliver the drug to psoriatic skin.

Recent works have also introduced transdermal aids such as the loading of liposomes into microneedles or pre-treating psoriatic lesions prior to liposome application to improve the transdermal flux of drugs to viable dermis. In Nakamura et al., 2024, iontophoresis, which is a physical intradermal drug delivery tool employing a small electric current that has been used to help drug permeation through the stratum corneum barrier, was used to assist drug delivery of liposomes [82]. In the imiquimod-induced skin model, TFH-based anionic liposomes loaded with tacrolimus were applied to iontophoresis-treated skin that has provided a transient suppression of TNF-α and IL-6 levels as well as lessening epidermal thickening than skin treated with conventional ointment, indicating liposomal delivery of drug could be further aided with an iontophoresis approach without compromising diseased skin. Qu et al., 2023 manufactured liposomes containing dexamethasone that were later size reduced by sonication and membrane extruded before being loaded onto a hyaluronic acid microneedle [83]. In the imiquimod-induced psoriatic model, cationic liposomes were more effective than anionic liposomes when delivered through the microneedle system in terms of improvement in histological features with decreasing levels of proinflammatory cytokines. This was contributed by better cellular uptake and skin retention based on results obtained using cationic liposomes containing fluorescence dye on psoriatic skin lesions. In Shen et al. 2024, liposomes that were produced by a reverse phase evaporation process were membrane extruded before chemically conjugated with a fixed ratio of hyaluronic acid [84]. The functionalized liposomal gel was then used to entrap methotrexate via a “small volume incubation (SVI)” procedure before loading onto hyaluronic acid-based microneedles. The modified liposomal gel in the microneedle delivered more drug via transcutaneous route than the drug-loaded gel alone based on an ex vivo rat skin model and the outcomes were complemented by results from the induced mice skin model with reduced PASI score, epidermal thickness, mRNA levels of IL-17A, IL-23 and TNF-α and proliferative cell-associated antigen Ki67 expression. TFH-based ginsenoside Rg3 liposomes that underwent size reduction by sonication were loaded onto microneedles made from polydimethylsiloxane and sodium hyaluronate [85]. In the imiquimod-induced skin model, microneedle tips that dissolved rapidly to release drug-loaded liposomes decreased the levels of IL-17, TNF-α and IL-23, proving the efficacy of such treatment. Hence, the benefit of transdermal tools in aiding the delivery of drug-loaded liposomes into psoriatic lesions has been demonstrated without adverse effects on skin conditions. In these cases, the vesicular size reduction step that may impact drug entrapment may be avoided when combined with transdermal delivery tools.

#### Liposomes with Combined Anti-Psoriatic Therapy

Patients are often prescribed multiple topical therapies that they have to apply separately to relieve symptoms related to psoriatic flare. To reduce the number of applications necessary to better control symptoms and to gain better compliance, topical therapy loaded with dual anti-psoriatic agents has been prepared, and there is a small number of marketed products such as Dovobet ointment and Enstilar foam containing fixed doses of Calcipotriol and Betamethasone Dipropionate. Nanocarriers are also used to entrap dual anti-psoriatic agents, where a nanostructured lipid particle is a popular option as it has shown a good storage stability profile and solvent capacity with good drug entrapment [86]. Utilizing liposomes to encapsulate multiple anti-psoriatic agents has also been reported. Table 1 summarizes recent research for liposomes loaded with combinational therapy of anti-psoriatic agents. In Wang et al., 2020, all-trans retinoic acid and betamethasone co-loaded TFH-based liposomes were produced using lecithin and Tween 80 and then sonicated before incorporated into carbomer gel [87]. Vesicles showed high entrapment efficiency at relatively low drug loading for both drugs. Studies using HaCaT cell line and imiquimod-induced mouse models indicated that liposomal gel lowered levels of IL-6 and TNF-α in a time-dependent manner based on cellular uptake rate. Elhabal and co-workers prepared cationic cerosomes resembling liposomes with Ceramide III, phospholipid and Kolliphor RH40 or TPGS as main membrane components [88]. The vesicles were manufactured by hydrating the dried lipid film in a hyaluronic acid solution to co-load ciclosporin and dithranol. In the imiquimod-induced psoriatic mice model, topical application of cerosomes containing dual drug agents has reduced PASI score when compared to Betnovate ointment, a commonly used topical steroid. In Chen et al., 2021, a combination of zedoary turmeric oil and tretinoin containing anionic liposomes was prepared using soy phosphatidylcholine and cholesterol before dispersing into Carbopol gel [89]. The entrapment efficiency of tretinoin at lower drug loading was higher than turmeric oil, with drug loading close to 10%. The liposomal gel showed gradual penetration of drugs into the hair follicles of mice models when compared to conventional gel. For the mice tail model, liposomal gel was more effective than conventional gel, and clinical improvement showed a dose-dependent effect on psoriasis. In another study, cationic liposomes consisting of soybean phosphatidylcholine and cholesterol as main lipid components were produced using the solvent injection method that exhibited high and similar entrapment efficiency at a 1:2 ratio of Ibrutinib and curcumin [90]. Liposomes were then dispersed in carbopol-940 gel and tested in the imiquimod-induced skin model. Liposomal gel containing dual drugs improved histological features such as decreased epidermal hyperplasia, PASI index and levels of IL-22, TNF-α, IL-17, IL-23 and IL-6 and IL-2 when compared with individual drug formulation and plain drug gel [90]. Co-loading dual agents into liposomes has shown synergistic drug effects to promote symptom relief and disease control in both in vitro and in vivo psoriatic models. In many cases, short-term storage stability of liposomes containing dual anti-psoriatic agents based on size distribution has been demonstrated. Charged or neutral liposomes can be manufactured based on the constituents of the lipid bilayer and the medium that these liposomes were immersed in. Types of drugs or surface-modulating agents added can modulate the surface properties of liposomes. As surface charge density can affect the long-term stability of liposomes, it may alter the drug entrapment efficiency as well as change the interaction of liposomes and cells of the skin. These aspects warrant a systematic investigation using suitable psoriatic skin or 3D cell culture models—Figure 2—in their final dosage forms.

### 5.2. Liposomes and Breast Cancer

In the past ten years, major advances in nanotechnology and novel drug carriers paved the road towards safer and more effective breast cancer treatment strategies compared with conventional modalities. Furthermore, advances in molecular biology and pharmacology aided in a better understanding of breast cancer, enabling the design of smarter therapeutics able to target cancer and respond to its microenvironment efficiently by providing the ‘selective’ delivery to the target area. The ideal solution would be to target the drug alone to those cells, tissues and organs that are affected by the disease [39,91].

Various liposomal-based formulations were successfully implemented in clinical fields as having antitumor properties. Doxil^®^ was the first approved clinical anticancer liposome doxorubicin (DOX) in the USA (1995). It opened the way for several other liposomal formulations to reach the clinical application fields by innovating the pH gradient active loading and usage of PEGylation for stealth liposomes [92]. Myocet^®^ is another liposomal-based, doxorubicin citrate-encapsulated formulation (180 nm and composed of 1-palmitoyl-2-oleoylphosphatidylcholine:cholesterol, mole ratio 55.8:44.2), but it lacks the PEGylated coating. It is characterized by a shorter circulation time when compared with Doxil^®^, with dramatically reduced cardiac toxicity [68]. Myocet^®^ was compared with free doxorubicin in preclinical toxicity studies performed on Beagle dogs, in which Myocet^®^ has shown a better toxicity profile than free doxorubicin [93].

Daunorubicin (DNR) is another anthracycline antibiotic (isolated from *Streptomyces peucetius varcaesitue*) with anticancer activity [68]. It works the same as DOX with significant side effects, such as cardiotoxicity (dose-dependent), alopecia, nausea and vomiting, which are associated with DNR therapy, but liposomal daunorubicin formulation has been developed as an alternative to reduce some of these adverse side effects. Onivyde™, an irinotecan (IRI) liposome injection, is a product of Merrimack Pharmaceuticals Inc., Cambridge, MA, USA, approved in 2015. Onivyde™, coupled with leucovorin and fluorouracil, is recommended for the management of patients with metastatic adenocarcinoma of the pancreas that has shown disease progression after gemcitabine-based therapy. Onivyde™ is formulated with a water-soluble semi-synthetic IRI hydrochloride trihydrate, a topoisomerase inhibitor, into a liposomal dispersion. Onivyde™ liposomes are unilamellar lipid bilayer vesicles with a mean diameter of 110 nm that encapsulates IRI in a gelated or precipitated state as the sucrose octasulphate salt using an ion-exchange/titration method in aqueous space. Onivyde™ was prepared using a novel method, i.e., intra-liposomal drug stabilization technology, which encapsulates drug molecules into long-circulating liposome-based nanovesicles. The vesicle is composed of DSPC, cholesterol and methoxy-terminated polyethylene glycol (MW 2000)-distearoylphosphatidyl ethanolamine (MPEG-2000-DSPE) in the ratio of 3:2:0.015, which encapsulated more than 90% of the drug [93]. Liposomal IRI was compared with free IRI using human colon (HT29) and breast (BT474) cancer xenograft models. Liposomal IRI showed significantly enhanced cytotoxic activity due to exponentially higher drug loading and extended drug retention in vivo [94]. A randomized, open-label NAPOLI-1 clinical trial was conducted on patients with metastatic pancreatic adenocarcinoma whose cancer had progressed after consuming the chemotherapeutic agent gemcitabine or a gemcitabine-based therapy demonstrated the efficacy and safety of Onivyde™. The patients in the study who consumed fluorouracil/leucovorin with Onivyde™ survived 6.1 months on average, compared with 4.2 months on average for patients who consumed either fluorouracil or leucovorin. In another study, patients who consumed fluorouracil/leucovorin with Onivyde™ had an average delay of 3.1 months in the amount of time required for tumor progression, compared with 1.5 months for those who consumed either fluorouracil or leucovorin [95].

In addition to the commercially available liposomes with encapsulation of only one anticancer drug, there are many studies explaining the effects of various liposomal formulations with individual therapies on breast cancer cells; hence, those studies will not be covered in this review. In contrast, there are sparse literature reviews on novel therapeutic approaches on liposomes that encapsulate combinational therapy for targeting breast cancer cells with enhanced therapeutic efficacy and minimal side effects. Here, we will concentrate on recent research works in developing liposomal combinational therapy that targets breast cancer and liposomes using combinational therapy for breast cancer, which are summarized in Table 1.

Several drug combinations have been co-loaded into liposomes and characterized for their potential applications in treating breast cancer. Paclitaxel and rapamycin have been shown to act synergistically in breast cancer treatment, but the conventional formulation of paclitaxel causes side effects that limit its clinical use. Both drugs also suffer from pharmacokinetic limitations, reducing their in vivo efficacy. Drug delivery systems, particularly liposomes, offered a promising alternative by co-encapsulating these drugs and improving their therapeutic index. In Eloy et al., 2016, a PEGylated liposomal formulation of Soy phosphatidylcholine/cholesterol/DSPE-PEG-2000 was developed to co-load paclitaxel and rapamycin, resulting in high encapsulation efficiency, a nanometric particle size, low polydispersity and near neutral zeta potential [96]. Fourier transform infrared spectroscopy (FTIR) and thermal analysis revealed the conversion of paclitaxel and rapamycin to more bioavailable molecular and amorphous forms, respectively. The PEGylated liposomes showed excellent colloidal stability and a slow, sustained release profile. The co-encapsulated liposomes exhibited greater cytotoxicity against the 4T1 breast cancer cell line when compared to free drugs, with synergistic effects when both drugs were co-loaded. Also, the formulation reduced the tumor volume in 4T1 tumor-bearing mice significantly better when compared to paclitaxel and rapamycin alone or combined in free form and to paclitaxel and rapamycin alone in liposomal form, suggesting its potential for clinical application in the treatment of breast cancer. In another study, Simvastatin (SIM), a lipid-lowering agent, was combined with Doxorubicin (DOX) to enhance its effect. Duarte et al. (2023) produced PEGylated liposomes co-loading doxorubicin and simvastatin at different molar ratios using a thin film hydration method, and their potentials in breast tumor treatment were examined in human breast cancer cell lines (MDA-MB-231, MCF-7, SK-BR-3) [97]. The main compositions of liposomes were 1,2-Dioleoyl-sn-glycero-3-phosphoethanolamine (DOPE), cholesterol hemisuccinate and DSPE-PEG-2000. Liposomes loaded with dual agents were less than 150 nm with a narrow size distribution. Entrapment of DOX was performed by transmembrane sulfate gradient using SIM-loaded liposomes, and DOX was found to be better entrapped than SIM with overall efficacy close to 100% and over 60%, respectively, when initial loading solutions of 1 to 2 mg/mL were used. The liposomes exhibited pH-responsive dissolution profiles with more DOX and SIM being released at pH 5 than pH 7.4. The greatest inhibitory effect on cell proliferation and induction of death on breast cancer cell lines was achieved when a molar ratio for DOX to SIM of 2:1 was used, as both drugs acted synergistically to show the potential of liposomes with combinatory therapy for breast tumor treatment.

Overcoming multidrug resistance (MDR) is a major challenge in cancer treatment, and the co-delivery of anticancer agents in a single nanocarrier represents a promising strategy. Resveratrol was co-encapsulated with paclitaxel (PTX) in a PEGylated liposome of phosphatidylcholine/DSPE-mPEG2000 to create a combination therapy for drug-resistant breast cancer [98]. The resulting liposomes had an average diameter of 50 nm and encapsulation efficiencies exceeding 50%. In vitro studies demonstrated that the composite liposomes generated significant cytotoxicity against drug-resistant MCF-7/Adr tumor cells, while in vivo studies revealed improved bioavailability and tumor retention of the co-encapsulated drugs. Systemic therapy with the composite liposomes effectively inhibited tumor growth in mice without increasing toxicity (*p* < 0.01). The study suggested that co-delivery of resveratrol and paclitaxel in liposomes may enhance the treatment of MDR tumors by improving drug retention and reducing systemic toxicity. Similarly, Ye et al. (2022) developed a co-delivery liposomal system that combines docetaxel and curcumin (CUR-DTX-L) to overcome multidrug resistance in breast cancer [99]. The cholesterol/soybean liposomes were created using the thin-film hydration method and exhibited a particle size of 208.53 nm and a polydispersity index of 0.055. High encapsulation efficiency was achieved, and the liposomal system demonstrated sustained drug release over 72 h. An in vitro release study and cell viability with CCK-8 assay using MCF-7 breast cancer cells showed that the co-loaded liposomes CUR-DTX-L had better sustained release effects and antitumor efficacy than free drugs. The in vivo studies using tumor-bearing mice confirmed that the liposomal formulation CUR-DTX-L provided the highest tumor suppression and tumor inhibition rate (66.23%) compared to liposomal curcumin alone, liposomal docetaxel alone, non-liposomal (curcumin + docetaxel), free docetaxel and free curcumin. In addition, no substantial weight loss was detected in mice treated with CUR-DTX-L after the ninth day of treatment, in contrast to animals treated with other groups, suggesting that CUR-DTX-L can mitigate the negative effects and systemic toxicity of other groups. In another study, rapamycin (RAP) and resveratrol (RSV) were co-encapsulated in liposomes (RAP-RSV-LIP) to explore their potential for breast cancer therapy [100]. Liposomes were prepared using a high-pressure homogenization technique and were characterized by their negative surface charge, an average particle size of approximately 100 nm, low polydispersity and high encapsulation efficiencies (58.87% for RAP and 63.22% for RSV). The liposomes showed excellent stability over 60 days and a prolonged drug release profile. In vitro, RAP-RSV-LIP liposomes were internalized by estrogen receptor-positive MCF-7 breast cancer cells, achieving 34.2% cellular uptake and exhibiting enhanced cytotoxicity compared to free drugs. These findings indicate that RAP-RSV-LIP liposomes have significant antitumoral potential for breast cancer treatment and may provide a promising therapeutic option for further clinical studies.

The benefits of co-administering nutraceutical or dietary supplements and chemotherapeutical drugs in cancer treatment were also explored for breast cancer treatment. Phosphatidylcholine/cholesterol-based liposomal formulation for co-delivery of docetaxel (DTX) with palmitoyl ascorbate (PA) was prepared [101]. Palmitoyl ascorbate (PA) has been chosen as it is an antioxidant that can stabilize liposomes and enhance anti-cancer effects. TFH-based anionic liposomes co-loaded with various weight ratios of DTX and PA were fabricated and then ultrasonicated to reach between 140 and 170 nm. These liposomes exhibited high encapsulation efficiencies of PA and DTX regardless of DTX:PA ratios, though drug ratios influenced the anti-cancer effects of HepG2, MCF-7 and PC-3 cells differently. In the MCF-7 breast cancer model, only liposomes with a DTX:PA ratio of 1:200 led to the highest anti-cancer activity, while other liposomes with smaller PA contents were less effective. The co-loaded PA/DTX liposomes could be subjected to in vivo tests to confirm efficacy for breast cancer. Alpha-linolenic acid (ALA) is an omega-3 fatty acid found in dietary supplements. It can dampen inflammatory signals and has anticancer properties. Kumar et al. (2024) produced ALA and Paclitaxel (PTX) co-loaded TFH-based anionic liposomes constituted of Phosphatidylcholine, cholesterol and D-alpha-tocopheryl polyethylene glycol 1000 succinate [102]. These liposomes had good entrapment of PTX following probe sonication. In the MCF-7 breast cancer cell line, ALA-PTX liposomes had a lower inhibitory concentration than ALA liposomes or PTX liposomes of similar drug entrapment efficacy. The beneficial effect of ALA-PTX liposomes was further confirmed by exhibiting greater morphological apoptotic changes in treated cells, suggesting enhanced anticancer efficacy that was further aided by an increased membrane fluidity induced by ALA. Pogorzelska et al. (2023) combined doxorubicin (DOX) and sulforaphane (SFN), a multipotent chemo-preventive and anticancer isothiocyanate with antioxidative properties from the *Brassicaceae* family, in a liposomal formulation [103]. The cationic nano-sized liposomes were produced by ultrasound sonication using 1,2-dimyristoyl-sn-glycero-3-phosphocholine (DMPC) as the main lipid. Using the MDA-MB231 cell model, DOX and SFN interacted synergistically, where up to a fourfold reduction in DOX concentration could still inhibit tumor growth. Accordingly, SFN increased the accumulation of DOX in the nuclei of cancer cells, inhibiting mitosis without affecting the reactive oxygen species status of the cell. This has indicated the protective benefit of SFN in ameliorating unwanted effects of DOX. The efficacy and safety of liposomes loaded with dual agents were further confirmed by the 4T1 mouse model of triple-negative breast cancer, where the histological and biochemical outcomes were found favorable after administration of liposomes with DOX/SFN, showing organ protective effects. A recent study explained curcumin-loaded liposomes modified with folic acid (LIP-CCM-FA) to enhance the treatment of breast cancer by targeting folate receptors overexpressed in tumor cells [104]. Both LIP-CCM-FA and liposomes with no folic acid (LIP-CCM) were prepared with DSPC, cholesterol and Polyethylene glycol 1000 succinate, showing a particle size of approximately 138 nm, a polydispersity index (PDI) around 0.14, a negative zeta potential (~−13 mV) and high encapsulation efficiency (>73%). In vitro tests using 2D and 3D MCF-7 breast cancer models demonstrated that LIP-CCM-FA had significantly higher cytotoxicity compared to free curcumin and LIP-CCM. The folic acid-modified liposomes also showed enhanced cellular uptake and spheroid penetration, indicating improved drug internalization. These results suggest that folic acid-modified liposomes could provide a targeted and more effective treatment for breast cancer.

Patel and co-workers explored the anti-cancer effect of combining mycophenolic acid (MPA) and quercetin (QC) in liposomal forms [105]. THF-based anionic liposomes loaded with either MPA (LNP-MPA) or QC (LNP-QC) were made of cholesterol and soya lecithin as membrane components, and these liposomes were probe sonicated to <200 nm. The entrapment efficiency for MPA was higher than QC regardless of initial drug loading, and all liposomes exhibited over 60% drug entrapment. Exposure to blended liposomes (LNP-MPA + LNP-QC) had led to a higher cellular uptake and cytotoxicity than either LNP-MPA or LNP-QC alone in the MCF-7 breast cancer model. Following intraperitoneal injection of liposomes in the rat model, the bioavailability of MPA was the highest with blended liposomes, and the anti-tumor effect of this combined therapy was better in the DMBA-induced breast cancer rat model exhibiting the biggest tumor mass reduction compared to non-liposomal MPA/QC, LNP-QC or LNP-MPA. Hence, co-administering liposomal blends loaded with different drugs to treat breast cancer can be an alternative approach to co-loading both agents into liposomes.

**Table 1:** Details the undertaken research for liposomes loaded with either anti-psoriatic or anti-breast cancer agents as combinational therapy.

Formulation/Method	Drug Candidates	Model	Study Outcomes	Reference
Psoriasis Treatment				
Flexible liposomes by thin-film hydration then dispersed in Carbopol gel	All-trans retinoic acid + Betamethasone	HaCaT cell line and imiquimod-induced mouse skin model	Dual agents loaded liposomal gel showed time-dependent cellular uptake and lowered levels of inflammatory cytokines more than single agent gel	[87]
Cationic cerosomes in hyaluronic acid by thin-film hydration	Ciclosporin + Dithranol	HSE-2 Cells, Ex-vivo mice skin and imiquimod-induced psoriatic mice skin model	Cerosomes lowered level of proinflammatory cytokines in cell culture, enhanced the skin penetration of both drugs by 66.7% compared to drug solution and reduced PASI score and cytokine levels when compared to marketed ointment and free drug preparations in vivo	[88]
Anionic liposomes by ethanol injection then dispersed in Carbopol gel	Zedoary turmeric oil + Tretinoin	Mouse vaginal and tail models	Liposomal gel showed gradual penetration of drugs into the hair follicles when compared to conventional gel. It inhibited mitosis of mouse vaginal epithelium and promoted the formation of stratum granulosum in mouse tail in a dose-dependent manner	[89]
Cationic liposomes by solvent injection then dispersed in Carbopol gel	Ibrutinib + Curcumin	Imiquimod-induced psoriatic mouse model	Better histological features, PASI index and lower levels of inflammatory cytokines when compared with individual drug formulation and plain drug gel	[90]
**Breast cancer treatment**				
PEGylated liposomes by thin-film hydration	Paclitaxel + Rapamycin	4T1 cell line, BALB/c mice	In vitro and in vivo results showed that liposomes co-loaded with both drugs had higher cytotoxic effect compared to the free drugs	[96]
PEGylated liposomes by thin-film hydration method.	Simvastatin + Doxorubicin	MDA-MB-231, MCF-7, and SK-BR-3 human breast cancer cell lines	Liposomes exhibited pH responsive drug release; co-loading Simvastatin and Doxorubicin in liposomal form significantly increased the inhibition of cell proliferation	[97]
PEGylated liposomes by film hydration method	Resveratrol + Paclitaxel	MCF-7/Adr tumor cells BALB/c nude mice	Liposomes showed a potent cytotoxicity against the drug-resistant MCF-7/Adr tumor cells in vitro, enhanced bioavailability and tumor-retention of the drugs in vivo. Systemic therapy effectively inhibited drug-resistant tumor in mice significantly without any notable increase in the toxicity	[98]
Anionic liposomes by ethanol injection method	Docetaxel + Curcumin	MCF-7 tumor-bearing mice	Liposomes co-loading dual agents showed the highest cytotoxicity, tumor volume reduction and with less systemic toxicity than liposome loaded with Docetaxel alone	[99]
Anionic liposomes by high-pressure homogenization method	Rapamycin + Resveratrol	MCF-7 breast cancer cell line	In vitro studies indicated that liposomes were internalized in an estrogen receptor-positive human breast cancer cell line and improved cytotoxicity when compared with free drugs	[100]
Anionic liposomes by thin-film hydration method	Docetaxel + Palmitoyl ascorbate	MCF-7 breast cancer cell line, liver (HepG2), and prostate (PC-3) cancer cell lines	Co-delivery of Docetaxel+ Palmitoyl ascorbate in the liposomal system enhanced the antitumor therapy when high ratio of Palmitoyl ascorbate was used	[101]
Anionic liposomes by thin-film hydration method	Paclitaxel + Alpha-Linolenic Acid	MCF-7 breast cancer cell line	Liposomes with dural agents showed enhanced cellular uptake and superior anticancer efficacy compared to liposomal Paclitaxel only.	[102]
Cationic liposomes by ultrasonication	Doxorubicin + Sulforaphane	4T1 mouse model of triple-negative breast cancer, MDA-MB-231 breast cancer cell line	Incorporation of sulforaphane resulted in a two-fold inhibition of tumor growth and up to a four-folds reduction in doxorubicin concentration. Sulforaphane was shown to increase the accumulation of doxorubicin in the nuclei of cancer cells	[103]
Folic acid-modified anionic liposome by thin-film hydration	Folic acid + Curcumin	MCF-7 breast cancer cell line (2D and 3D cell culture models)	Significant cytotoxicity effect and higher cellular internalization compared to free curcumin and liposomal curcumin	[104]
Anionic liposomes loaded with single agent by thin-film hydration. Combined therapy was blend of two types of liposomes	Mycophenolic Acid + Quercetin	MCF-7 breast cancer cell line, Sprague-Dawley rat model	Combination of liposomal (Mycophenolic Acid) + liposomal (Quercetin) showed higher cellular uptake, cytotoxicity and anti-cancer effect compared to individual formulation or free drugs	[105]

## 6. Conclusions

Liposomes are spherical-shaped vesicles consisting of one or more phospholipid bilayers. Liposomes have been proven as a suitable candidate for drug delivery in nanomedicine due to their small vesicle size, biocompatibility, nontoxic, non-immunogenic, and biodegradable natures. High cellular uptake, improved efficacy, reduced drug dose and easy functionalization ability are other good features of liposomes; in addition to their capacity for surface modifications for targeted, prolonged and sustained release, liposomes can target cells (e.g., psoriatic cells and breast cancer cells) via enhanced permeability and retention effect process. Many liposomal-based drug delivery systems are currently clinically approved to treat several diseases such as cancer of various types and viral infection. In this review, innovative liposomes with combinational therapy have been literature proven to overcome the limitations of liposomes with monotherapy to treat psoriasis or breast cancer since the incorporated drugs interact in a synergistic manner to potential therapeutic effect, to lower side effects and/or stabilize liposomes. They were found effective when administered either topically or parenterally for psoriasis and breast cancer, respectively, and may be applicable to other routes of administration.

Both breast cancer and psoriasis involve inflammatory cytokines such as TNF-α and IL-17/IL-23 axis, which play critical roles in immune system regulation [6,44]. There were case studies showing cancer treatments making patients’ existing psoriasis worse; for example, docetaxel and immunotherapy triggered psoriasis ‘flare-ups’ in prostate and lung cancers, respectively [106,107]. A recent study based on transcriptome analysis has suggested a co-morbid mechanism existed for breast cancer and psoriasis and the study further indicated an increased risk of developing certain breast cancer in psoriasis patients [108]. In addition, a systematic review and meta-analysis highlighted that individuals with psoriasis have a higher risk of cancer incidence and a greater risk of cancer-related mortality [109,110], and prescribing sub-optimal therapy might be a contributory factor to balance the drug response on the body inflammatory pathway in these patients. This increased risk underlines the importance of studying these diseases together, particularly when considering innovative treatments like liposomal drug delivery systems loaded with combinational therapy that can target the disease and the pathways involved in both conditions. In summary, liposomes with combinational therapy are feasible to be taken into further investigations and clinical trials in the future.
